# Data in support of quantitative proteomics to identify potential virulence regulators in *Paracoccidioides brasiliensis* isolates

**DOI:** 10.1016/j.dib.2015.09.001

**Published:** 2015-09-09

**Authors:** Alexandre Keiji Tashima, Daniele Gonçalves Castilho, Alison Felipe Alencar Chaves, Patricia Xander, André Zelanis, Wagner Luiz Batista

**Affiliations:** a Departamento de Bioquímica, Escola Paulista de Medicina, Universidade Federal de São Paulo, São Paulo, SP, Brazil; b Departamento de Microbiologia, Imunologia e Parasitologia, Escola Paulista de Medicina, Universidade Federal de São Paulo, São Paulo, SP, Brazil; c Departamento de Ciências Biológicas, Universidade Federal de São Paulo, Campus Diadema, Diadema, SP, Brazil; d Instituto de Ciência e Tecnologia, Universidade Federal de São Paulo, Campus São José dos Campos, Rua Talim, 330, São José dos Campos, SP, Brazil

**Keywords:** *Paracoccidioides brasiliensis*, Pathogenic fungus, Proteomic analysis, Virulence factors

## Abstract

*Paracoccidioides* genus are the etiologic agents of paracoccidioidomycosis (PCM), a systemic mycosis endemic in Latin America. Few virulence factors have been identified in these fungi. This paper describes support data from the quantitative proteomics of *Paracoccidioides brasiliensis* attenuated and virulent isolates [1]. The protein compositions of two isolates of the Pb18 strain showing distinct infection profiles were quantitatively assessed by stable isotopic dimethyl labeling and proteomic analysis. The mass spectrometry and the analysis dataset have been deposited to the ProteomeXchange Consortium via the PRIDE partner repository with identifier PXD000804.

## Specifications Table

**Table:** 

Subject area	*Biology*
More specific subject area	*Microbiology, proteomics*
Type of data	*Mass spectrometry data and worksheets with peptide and protein identifications.*
How data was acquired	*Data dependent LC-MS/MS acquired in LTQ-Orbitrap Velos (Thermo) coupled to EASY-nLC II (Thermo).*
Data format	*RAW files from the instrument and.csv files exported from PEAKS Studio 7 (Bioinformatics Solutions Inc.).*
Experimental factors	*Attenuated and virulent Paracoccidioides brasiliensis (Pb18) strains were grown in minimal and rich media for comparative proteomic analysis.*
Experimental features	*Pb18 protein extracts were digested with trypsin and peptides labeled by isotopic dimethylation.*
Data source location	*Diadema and São Paulo, Brazil.*
Data accessibility	*Mass spectrometry and proteomics data available via ProteomeXchange Consortium with identifier PXD000804*

## Value of the data

• A new approach to identify potential virulence regulators in *P. brasiliensis* using the same isolate (Pb18) with different degree of virulence through quantitative proteomics.
• A comprehensive protein list of attenuated and virulent *P. brasiliensis* (Pb18) strains grown in minimal medium (MM) and rich medium (RM).
• A large dataset of proteins involved with virulence regulation in *P. brasiliensis* identified by stable isotopic dimethyl labeling-based mass spectrometry, which were validated by quantitative PCR assays.

## 1 Data, experimental design, materials and methods

**Fig. 1:**
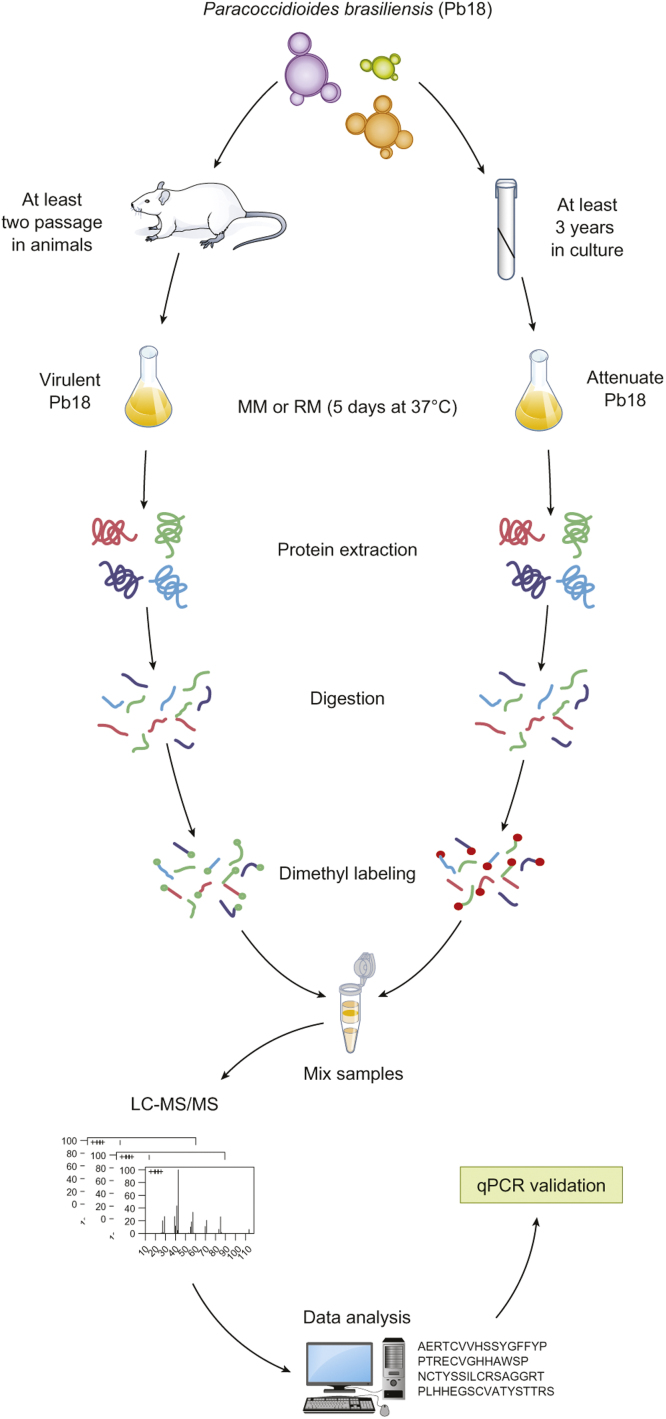
Schematic representation of the experimental strategy for proteomic analysis of alterations in *Paracoccidioides brasiliensis* (isolate Pb18) with different degrees of virulence. Virulent Pb18 was obtained by passage in animals and attenuated Pb18 was reached by successive sub-culturing in vitro. Before protein extraction, virulent and attenuated Pb18 were grown in MM or RM for 5 days. Then, the proteins were extracted, denatured with 4 M GuHCl, reduced with 5 mM DTT, alkylated with 15 mM iodoacetamide and digested separately with trypsin. Tryptic peptides were differentially labeled via stable-isotope dimethyl labeling. Samples (vPb18 and aPb18) were mixed, desalted and dried. Peptides were redissolved in formic acid and subjected to nanoLC system coupled with LTQ-Orbitrap mass spectrometer for MS analysis. Raw data were acquired by Xcalibur software, and were searched against the *Paracoccidioides brasiliensis* strain Pb18 database using PEAKS Studio 7. qPCR assays were performed to validate the data.

Using a quantitative shotgun proteomic approach (nLC–ESI–MS/MS) we have previously identified in these dataset proteins with potential role in the regulation of virulence in the pathogenic fungus *Paracoccidioides brasiliensis* [1]. The experimental workflow was designed to compare attenuated and virulent Pb18 strains in MM or RM media, to identify potential proteins involved in the virulence regulation. Each strain was grown in biological duplicates, proteins were extracted, digested with trypsin, labeled with light and heavy formaldehyde, and each biological replicate was analyzed by LC–MS/MS in technical triplicates. Raw data were processed and analyzed in PEAKS Studio 7.0 (Bioinformatics Solutions Inc.) and proteins were quantified by the ratios of heavy/light peptide intensities. qPCR assays were performed to validate the LC–MS/MS data (Fig. 1).

### 1.1 Fungal strain and growth conditions

We used *P. brasiliensis* Pb18 isolates (with different degrees of virulence) in our experiments. Yeast cells were cultured and maintained at 37 °C in modified YPD (modYPD) medium (0.5% yeast extract, 0.5% casein peptone, and 1.5% glucose, pH 6.5). After 3 passages on solid medium, the virulent Pb18 (vPb18) isolate was used to infect mice (B10.A) and then re-isolated. The attenuated isolate (aPb18) was maintained in culture media (modYPD) at 37 °C for at least 3 years. This concept of attenuation or loosing virulence of *P. brasiliensis* by long-term cultures was initially described by Kashino and co-workers [2] and Brummer and co-workers [3]. To recovery fungi virulence of aPb18, Balb/c mice were intraperitonealy (i.p.) infected with 1×10^6^ viable yeast cells of aPb18 (attenuate form) in 50 µL of phosphate-buffered saline (PBS). After 20 days, the mice were euthanized and the microorganisms in the lungs, liver and spleen were aseptically recovered. For this, each organ was removed, weighted and macerated in 5 mL of PBS. After, 100 μL of solution contained fungal cells was plated in BHI medium (supplemented with bovine fetal serum and growth factors). The plates were incubated at 37 °C for 10 days. The growth yeast cells were used to re-infect mice. All procedures describe above was repeated once more.

### 1.2 Protein extraction

Proteins were extracted from *P. brasiliensis* (vPb18 and aPb18 isolates) according to the protocol of Villén and co-workers [4] with some modifications. Yeast cells were grown to mid-log phase in modYPD (rich medium – RM) or Minimum Medium (poor medium – MM) for 5 days at 37 °C. Cells were collected and pelleted by centrifugation ( 2000*g*, 15 min, 4 °C), rinsed 3 times with cold PBS, distributed into 2 mL tubes and subjected to centrifugation at 10,000*g* for 5 min at 4 °C. The supernatant was subsequently removed, and cytoplasmic proteins were prepared by homogenizing the yeast cells with glass beads (beads 425–600 μm-Sigma, St. Louis, MO, USA) in 700 μL of cold lysis buffer (50 mM Hepes (pH 7.5), 2 mM EDTA, 2 mM DTT, 50 mM KCl, 0.2% Triton X-100, 1 mM sodium orthovonadate, 1 mM PMSF, 10 mg/mL aprotinin and 10 mg/mL leupeptin). Then, the yeast cells were mechanically disrupted using a Mini BeadBeater (Biospec Products, Bartlesville, OK, USA) at 4 °C (4 cycles of 90 s, with 60-s rest in between) and centrifuged at 1000*g* for 3 min at 4 °C to separate the glass beads from the lysate. The supernatant was collected and centrifuged at 15,000*g* for 10 min at 4 °C. The protein concentration in the supernatant was determined by Bradford protein assay (Bio-Rad Laboratories, Hercules, CA, USA). The samples were aliquoted and stored at −80 °C.

### 1.3 Sample processing protocol

Protein reduction, alkylation and enzymatic digestion proteins were processed according to the protocol of Kleifeld and co-workers [5] with some modifications. Briefly, 200 µg of protein from each sample (vPb18 and aPb18) was denatured with 4.0 M GuHCl (guanidine hydrochloride). Following the reduction of disulfide bonds with 5 mM dithiothreitol (DTT) for 1 h at 65 °C and alkylation of cysteine with 15 mM iodoacetamide (IAA) for 1 h at room temperature in the dark, the proteins were precipitated with 8 volumes of cold acetone and dried in a SpeedVac (Thermo Scientific, Bremen, GA, USA). The proteins from the different groups were subsequently dissolved in 5 µL of 100 mM NaOH and digested with 2 µg of sequencing grade modified trypsin (Promega, Madison, WI, USA) in 195 μL of 50 mM HEPES buffer (pH 7.5) (1:100, trypsin:protein) overnight at 37 °C.

For the stable isotope dimethyl labeling, the tryptic peptides (pH 6–7) from each sample (vPb18 and aPb18 cultivated in MM or RM) were separately reconstituted with 1 M NaBD_3_CN (to a final concentration of 20 mM) and incubated overnight at 37 °C. Samples were labeled with 2 M ^12^CH_2_O (light formaldehyde) for the aPb18 samples or ^13^CD_2_O (heavy formaldehyde) for the vPb18 samples to a final concentration of 40 mM light/heavy formaldehyde, resulting in mass differences of +30.04 and +36.08 Da for the light and heavy formaldehyde, respectively, for each completely labeled N-terminal or Lys site. We repeated the labeling with light or heavy formaldehyde (to strengthen the labeling) via incubation for an additional 2 h at 37 °C with 1 M NaBD_3_CN (to a final concentration of 10 mM) and labeling with 2 M ^12^CH_2_O or ^13^CD_2_O at a final concentration of 20 mM. To quench the reaction, 1 M Tris (pH 6.8; to a final concentration of 200 mM) was added to each sample and incubated for 2 h at 37 °C. Then, the labeled samples with the light and heavy isotopes were combined (1:1) and desalted using StageTips (C18-SCX-C18) according to the published protocol provided by Rappsilber and co-workers [6]. After desalting, the samples were finally dried in a SpeedVac and redissolved in 0.1% formic acid prior to nano-LC-MS/MS analysis.

### 1.4 Mass spectrometric analysis

A LTQ-Orbitrap Velos mass spectrometer (Thermo Fisher Scientific, Bremen, GA, USA) coupled with an Easy-nLCII (Thermo Fisher Scientific, Bremen, GA, USA) was used for comparative proteomic analysis. All columns were packed in house. The trap column (50 mm×100 μm I.D.) was packed with Jupiter C18 resin (10 μm, Phenomenex Inc, Torrance, CA, USA) and the analytical column (100 mm×75 μm I.D.) was packed with ACQUA C18 resin (5 μm, Phenomenex Inc, Torrance, CA, USA). The sample was delivered to the trap column at 2 μL/min in 100% solvent A (0.1% formic acid (Sigma, St. Louis, MO, USA)). Solvent B consisted of 0.1% formic acid in acetonitrile. Gradient elution was as follows: 7–45% solvent B over 160 min; 45–85% solvent B over 15 min; 85% solvent B for 3 min; and then a return to the initial condition (7% solvent B over 13 min), at a flow rate of 200 nL/min. The source was operated in positive ionization mode, with the voltage and temperature adjusted to 1.8 kV and 200 °C, respectively. The mass spectrometer was programmed in data-dependent acquisition mode, in which a scanning mass in the region of 350–1600 Th was employed (with a target value of 10^6^ ions) using the LTQ-Orbitrap analyzer with a resolution of 60,000 (in *m*/*z* 400), followed by collision-induced dissociation (CID using the ion trap analyzer) of the 10 most intense ions, with a dynamic exclusion time of 90 s. The window for the isolation of precursor ions was set to 2 Da, and the minimum count of ions to trigger events (MS2) was 10,000. The raw MS data were generated by Xcalibur version 2.2 (Thermo Fisher Scientific, Bremen, GA, USA).

### 1.5 Data analysis

The MS raw data was processed and searched in PEAKS Studio 7 (Bioinformatics Solutions Inc, Waterloo, Canada) against the *P. brasiliensis* strain Pb18 database (8741 entries, *P. brasiliensis* Sequencing Project, Broad Institute of Harvard and MIT, http://www.broadinstitute.org/) with added dimethyl masses. The following parameters were set for the PEAKS searches: cysteine carbamidomethylation was selected as a fixed modification, whereas methionine oxidation, acetylation of protein N-terminal, the deamidation of Asn/Gln and light or heavy dimethyl labeling of N-terminal and lysines (+30 Da and +36 Da, respectively) as variable modifications. The precursor and fragment ion tolerance were set as 15 ppm and 0.5 Da. Two missed cleavages were allowed for trypsin and a maximum of four variable PTMs per peptide. Relative quantification data were evaluated by PEAKS Q. The quantified proteins were accepted if they contained both light and heavy formaldehyde labeling and over 1 single peptide for protein. Proteins ratios were calculated as heavy/light formaldehyde intensities. Differentially expressed proteins were considered for ratios ≤0.5 (down-regulated) and ≥2 (up-regulated). Data from this work may be accessed from PRIDE (http://www.ebi.ac.uk/pride/archive/).

### 1.6 *Paracoccidioides* RNA isolation and analysis

*Paracoccidioides* yeast cells grown to exponential phase were collected by centrifugation ( 2000*g*), washed 4 times with PBS and resuspended in TRIzol reagent (Invitrogen, Carlsbad, CA, USA). RNA was isolated from yeast cells by mechanical disruption by vortexing with 0.5 mm-diameter glass beads for 10 min (10 cycles of 60 seg, with 60-seg rest in between). RNA was purified through extraction with CHCl_3_, followed by alcohol precipitation of the aqueous phase. Contaminating DNA in these preparations was digested with RNase-free DNase I (Promega). Briefly, RNase-free DNase treatment was done in a final volume of 10 μL containing 40 mM Tris–HCl (pH 8.0), 10 mM MgSO_4_, 10 mM CaCl_2_, 2 μL of RNase-free DNase (1 U/μL; Promega) and 2 μg of total RNA. The reaction was incubated for 30 at 37 °C min and stopped by incubation with 1 μL EGTA (20 mM pH 8.0) for 10 min at 65 °C. The absence of DNA contamination after the RNase-free DNase treatment was verified by PCR amplification of the Pb*GP43* gene (Accession No. U 26,160). Total RNA was reverse transcribed using a RevertAid Premium Reverse Transcriptase kit (Thermo Scientific, Bremen, GA, USA) using 2 µg of total RNA and oligo-dT 22-mer primers, according to the manufacturer's instructions.

qPCR assays was conducted using SYBR-Green (Applied Biosystems, Thermo Fisher Scientific Brand, Foster City, CA, USA). The reactions were performed using ABI Prism 7500 Sequence Detection System (Applied Biosystems) with equal amounts of each cDNA. A 10-μL total volume was used for each PCR reaction, which consisted of 1×SYBR Green PCR Master Mix, 250 nmol of the reverse primer, 250 nmol of the forward primer and 2 μL cDNA. The cycling parameters were 50 °C for 10 min, 95 °C for 5 min and 40 cycles at 95 °C for 30 s and 60 °C for 1 min. A non-template control (NTC) was used to detect any contamination. The dissociation curve was determined with an additional cycle of 95 °C (15 s), 60 °C (20 s) and 95 °C (15 s). After evaluating the quality of the reaction using dissociation curves, the data were analysed using ABI Prism 7500 software (Applied Biosystems). A threshold cycle value (Ct) was determined as the point at which the fluorescence exceeded the threshold limit. Amplification efficiencies were determined by comparing the dilution series of the reference gene and target gene from a reference cDNA template. The serial dilutions were amplified, and Ct values were obtained and used to construct a standard curve for each gene. The amplification efficiency was calculated using the following equation: *E*=10(−1/slope)−1, in which ‘*E*’ is the efficiency and ‘slope’ is the slope of the standard curve. A validation calculation was performed to evaluate if the efficiencies of the target gene and endogenous gene were approximately equal (90%≤*E*≤110%). The efficiencies of all genes analyzed were within this range. The relative transcript ratio (experimental/control) was determined based on the 2-[Delta][Delta]*C*_t_ method [7] after normalization with two housekeepers (*α-TUB* – PADG_01468 and *ALDH* – PADG_05081). Each reaction was performed in triplicate. Differences in the relative expression levels of genes were analyzed by defining reference cDNA control as the reference sample and setting its average value to 1. Fold-change values were logarithmized to the base 2 (log_2_). The data were expressed as the mean±SD, and *p* values were determined using Student's test.
